# Finding and Maintaining Motivation in Medical School

**DOI:** 10.31729/jnma.7521

**Published:** 2022-06-30

**Authors:** Rit Shrestha, Anjana Bohaju, Sarjan K.C., Anil Pathak

**Affiliations:** 1 Nepal Medical College and Teaching Hospital, Jorpati, Kathmandu, Nepal; 2 Kathmandu Medical College and Teaching Hospital, Sinamangal, Kathmandu, Nepal; 3 KIST Medical College and Teaching Hospital, Imadol, Lalitpur, Nepal

**Keywords:** *cognition*, *medical students*, *motivation*, *self-concept*

## Abstract

Medicine is a line of work in which expectations run high, having a competitive spirit is considered a virtue and relentless hard work is deemed a reward in itself. Yet, in a strenuous environment such as this, communication about the struggles medical students face is found to be almost nonexistent. This leads to medical students feeling burnt out, lost and inadequate, bringing about a drop in performance quality and/or quantity, which breeds further insecurity. This acts as a vicious cycle that is further perpetuated by the lack of effective communication, which becomes a bedrock for the deficit in support from peer groups, educators and authorities. There are different theories of motivation and these form the basis for exploring the different ways in which it can be increased. These methods, which contribute to enhancing productivity and curtailing stress, are detailed in the article.

## INTRODUCTION

As ambitious, hopeful young adults entering the vast expanse of medical education, we have often found ourselves questioning our intellectual capacities, passions and worth based on our academic proficiency and achievements. Almost every student who has been accepted into medical school is deemed quick on the uptake and brilliant- a perception that when coupled with an earnest desire to excel and achieve perfection, produces a state of mind that is more liable to selfdoubt, distress and anxiety when things go south. Medical students may feel like their attempts at merit are insufficient or that their ability to grasp information has reached a snag. The constant, nagging feeling of not being able to do or accomplish as much as they could have causes them to either overcompensate by working to the point of burnout or to stop trying all together. Students often report feeling lost and devoid of motivation, which is compounded by the highly competitive environment in which they are trained, the vast curriculum that they're expected to cover in a limited time frame, socioeconomic pressures and the high standards that their circle holds them up to. It causes them to question their passions, doubt their inclination towards medicine, devalue their self-worth and lose sight of their long-term goals, which further sends them down a path of mental distress. This manifests as depression, anxiety, burnout, fatigue, potentially substance abuse and decrease in quality of life.^1^

The psychological morbidities prevalent among medical undergraduates are significant, with the initial years of training being more psychologically draining due to the various stressors that act as assailants to a mind that is only recently learning to adapt to the exhaustive schedule of medical school.^2^ In the article, we have included the different factors that contribute to this grave situation (characterized by an amalgamation of feelings like remorse, discontentment and lack of fulfillment) that students of medicine experience in their undergraduate years and the ways in which productivity as well as mental wellbeing can be enhanced.

## MOTIVATION IN MEDICAL SCHOOL

Motivation forms the basis for action, confidence and ultimately, achievement. It can be considered the holy grail of productivity for students, especially ones of medicine. Evidence has shown that motivation for learning is something that exists and is significant as both a dependent and an independent variable: implying that it is both governed by the type of educational activity in question and also governs the quality of the learning experience.^3^ As learning materials pile up and objectives seem bleak, medical students often experience that their levels of inspiration and enthusiasm have plummeted. There are five theories of motivation: attribution theory, social cognitive theory, expectation value theory, goal orientation theory and self-determination theory.^3^

## A SYNOPSIS OF THE THEORIES OF MOTIVATION

### Attribution Theory

A medical student who has failed her anatomy exam may attribute her failure to a lack of effort or ability, while her peers may ascribe it to the fact that the exam was tough or that the examiner was biased. Opinions and causes that are assigned to an outcome vary among individuals and attributions do have an effect, albeit indirect, on the drive an individual has towards accomplishing a task.^3^

### Social Cognitive Theory

One student may flourish in an environment that is demanding and stressful, and find himself motivated to learn and grow, while another may realize that it seriously impacts his mental well-being and impedes his learning-the same learning environment can be motivating to one student while detrimental to another. Moreover, the belief one has on their own capabilities i.e., self-efficacy, which has little to do with their actual caliber, is equally crucial.^4^

### Expectancy Value Theory

This theory adds to the former in that it explains motivation as requiring more than just faith in the idea of achievement but also some personal gratification or worth. It connects motivation to the cumulative effects of expectancy of success and task value.^5^ Various factors contribute to either elevate or diminish the value of a task in the opinion of the learner and this consequently affects the motivation to persevere in the learning activity.

### Goal Orientation Theory

Learners tend to commit to an activity with the intention of either mastering the content (mastery goal), or the desire for doing better than others (performance-approach goals) or of avoiding failure (performance-avoidance goals).^6^ The former stems from a longing to learn while the latter two are more of success-centered/failure-dodging approaches. Evidence has shown that adopting mastery goals enhance both academic performance and well-being while performance-approach goals are linked to better academic performance but reduction in academic well-being. Learners with performance-avoidance goals however, had reduced academic performance as well as well-being.^6^ This shows how our ambitions are directly related to the level of our achievement and thus, motivation.

### Self-determination Theory

Each individual desires to feel autonomous, competent and connected to their social surroundings in order to be resolute in their actions.^3^ Students enter medical school with a desire to learn more about the human body or with a desire to train to become a successful doctor, but as the pressures that inadvertently accompany their education surmount, their motivations start becoming bleak. They are now replaced by action that is driven by deadlines, fear and self-imposed ambitions-factors that are crucial in their own right, but they certainly do not form the foundation for long lasting motivation. As reality becomes incongruent to expectations and the “why" becomes hazy, it is only normal that students experience a drop in inspiration, enthusiasm and ultimately, performance. This theory reinforces the need for identifying and holding on to the core reasons one has for performing a task, and also poses an interesting question: do situations have more power over our drive or does the way we view/handle them have more of an upper hand?

## FINDING MOTIVATION AND STAYING MOTIVATED

These theories exhibit significant overlap and each lead to a bigger picture-they help us see how motivation is an attribute that can expand and diminish depending on several factors. Each of these theories is a gateway that opens into the plethora of possibilities that will enable us to understand how we can build motivation and promote productivity among medical students. Here are a few strategies that can be assistive:

### Breaking the Cycle of Blame

Due to the differences in perspective, each student either considers negative consequences a result of their intrinsic shortcomings or that of external situations that are less than ideal. In either case, it is easy to get stuck in a loop of self-blame or a cycle of denial and ascribing fault. Instead of assigning blame to situations or individuals, it is with experience we state that finding the right balance between introspection and objectivity is integral to truly embracing the reasons why we feel like we are not experiencing the zeal that we desire and/or achieving the results we wish or expect to receive. For instance, observing the factors that influence our drive, coming to terms with our deficiencies (and those of our environment), maintaining accountability of our actions and tailoring our activities or study habits based on a sense of responsibility (academic, social, psychological) acted as crucial factors for understanding and eventually, building motivation.

### Raising the Bar for Success

We often experience that an activity we consider achievable ends up being more convenient to complete than one that we consider beyond our caliber. How often have we left a task undone because we believed it was just too difficult? These instances are a common occurrence, aided by the need for procrastination. However, actively tracking our progress and also recognizing previous achievements, all the while building resilience (by taking care of our physical, mental and emotional health) have been conducive to building sustainable motivation. The faith we have in our ambitions and in our capacity to achieve governs the enthusiasm we experience to actually pursue them and this is a notion that can enable us to find strength where we require it the most. This belief, along with the diligence we employ in monitoring our progress, has been very helpful to us.

### Reframing our Goals

The desire we have to pursue an activity depends on the reason why we choose to do it in the first place. Since mastery goal approach has been found to be far more superior in terms of ensuring academic wellness as well as excellence, choosing to learn something or to do an activity with the goal of becoming better instead of overtaking someone else (or worse, with the fear of falling behind), will ensure that our motivation levels are maintained. Some students have a habit of regularly checking in with themselves and internalizing the values that push them towards their goals- a strategy that is undoubtedly helpful. Are we studying to pass the test, to get a grade that is better than that of our peers or to actually gain command over the subject? We experienced that choosing the latter was certainly a better way of setting goals, with motivation levels, efficiency and the quality of learning being improved as a result.

### Recognizing Limits

When pursuing goals, internalizing the fact that there certainly are limitations to what can be achieved and learnt in a given time frame has been helpful. Exams become a time for students to bite off more than they can chew, with pressures mounting high. Setting realistic expectations and making attempts at completing attainable tasks help curb the loss of motivation that medical students experience when workloads are too high. This encompasses learning to say no to certain activities to be able to commit to the ones that bring value to us. As medical students, our interests are undoubtedly varied and the time we have is limited.

Focusing on the task at hand, which helps one hone their knowledge and gain skills, has been beneficial to us, for it has ultimately led to an increase in our faith when it comes to the prospect of success.

### Bringing a Change in the External Environment

External factors that influence motivation, though deemed to be less influential than intrinsic factors, cannot be slighted.^3^ For instance, early exposure to clinical cases can provide attainment value to the knowledge being imparted in the classroom, a strategy that is being implemented across medical colleges in Nepal. Being able to proactively correlate the knowledge that is imparted in the classroom with practical applications, has enabled most of us to strengthen our ties with the knowledge. Similarly, promoting effective interpersonal communication between students and teachers can help medical students thrive even in stressful environmentsa cooperation-based learning environment plus a work culture that is positive and open to hearing and seeking solutions to struggles of students would be far more conducive to learners.

Bringing about changes in our private milieu can be equally significant-keeping ourselves in touch with peers who are supportive and growth-oriented, maintaining a space that is exclusive to learning, scheduling our tasks and keeping visual reminders of things we feel good about has been helpful.^7,8^

### Preventing Physical and Mental Burnout

A dip in motivation is often accompanied by a feeling that our physical, mental and emotional resources have been spent, which is commonly known as burnout. Since we live in an environment that is laden with tension, it often becomes convenient to ignore our health. Although the stressors that influence mental health and wellbeing go beyond factors that are modifiable, the significance of changing elements that can be regulated cannot be slighted. Research has shown that consuming a healthy and balanced diet, regularly performing physical activity and getting restful sleep can be positively correlated with better outcomes in the realm of physical and mental health.^11^ By choosing to forego processed and “on the go" options for food, engaging in moderate to vigorous exercise and prioritizing sleep over all-nighters, we can make ourselves more productive.^9-12^ We have experienced this in our academic and personal lives- a healthy and well-rested body fosters a clearer mind; better sleep equals better focus, a better diet and regular exercise translates to a stronger body.

### Seeking Help When Needed

Although medical school can act as both precipitator and aggravator for psychological issues, there is no denying that help should be available and accessible to individuals who suffer from them. Chronic loss of motivation may be a symptom of a deeper underlying issue for example, depression; which may require diagnosis and treatment. We often wish to showcase our brilliance but tend to keep our struggles to ourselves. It is important that we normalize seeking help and embrace the fact that although struggle and strife is part of becoming a doctor, no one has to feel isolated. Being able to take advantage of affordable, confidential counseling services and mental health help can be a blessing to medical students. Besides these, a platform where students can share their experiences and find peers and colleagues, they can relate to on more than just a professional level should be encouraged. We have found these to be extremely helpful, especially when stressors were getting unbearably difficult to manage and motivation levels were low; a combination of dependable sounding boards and support systems during exams was indeed a boon!

## WAY FORWARD

Motivation is the foundation for effective action and gratifying achievement, both of which are prerequisites for success in medical school. Motivation for learning is a factor that can be built and nurtured using tried and tested techniques that have been detailed in the article. By learning about the theories of motivation and making sure that we internalize the solutions that they shed light upon, we can choose to be active participants in magnifying our productivity. As a process that is by nature, ongoing and continuous, staying on track would require diligence and patience-qualities that are crucial for future leaders in healthcare and education.
